# Safety, tolerability, and efficacy of metformin extended-release oral antidiabetic therapy in patients with type 2 diabetes: An observational trial in Asia

**DOI:** 10.1111/j.1753-0407.2012.00220.x

**Published:** 2012-12

**Authors:** Chul-Hee Kim, Kyung-Ah Han, Han-Jin Oh, Kevin Eng-Kiat Tan, Radhakrishna Sothiratnam, Askandar Tjokroprawiro, Marcus Klein

**Affiliations:** 1 Soonchunhyang University Bucheon HospitalGyeonggi-do; 2 Eulji General HospitalSeoul, Korea; 3 Cheil General Hospital & Women’s Healthcare CenterSeoul, Korea; 4 Mount Elizabeth Medical CentreSingapore; 5 Columbia Asia HospitalNegeri Sembilan, Malaysia; 6 Internal Medicine Department, Airlangga University/Dr. Soetomo General HospitalJawa Timur, Indonesia; 7 Merck Pte Ltd, Nordic European CentreSingapore

**Keywords:** Asian, extended-release, gastrointestinal side-effects, metformin, type 2 diabetes mellitus

## Abstract

**Background:**

The aim of the present prospective observational study was to assess the tolerability and antihyperglycemic efficacy of metformin extended-release (MXR) in the routine treatment of patients with type 2 diabetes mellitus (T2DM) from six Asian countries.

**Methods:**

Data from 3556 patients treated with once-daily MXR for 12 weeks, or until discontinuation, were analyzed.

**Results:**

Treatment with MXR was well tolerated, with 97.4% of patients completing 12 weeks of treatment. Only 3.3% of patients experienced one or more gastrointestinal (GI) side-effects and only 0.7% of patients discontinued for this reason (primary endpoint). The incidence of GI side-effects and related discontinuations appeared to be considerably lower during short-term MXR therapy than during previous treatment (mean 2.71 years’ duration), most commonly with immediate-release metformin. A 12-week course of MXR therapy also reduced HbA1c and fasting glucose levels from baseline.

**Conclusions:**

The present study provides new insights into the incidence of GI side-effects with MXR in Asian patients with T2DM and on the tolerability of MXR in non-Caucasian populations. Specifically, these data indicate that once-daily MXR not only improves measures of glycemic control in Asian patients with T2DM, but also has a favorable GI tolerability profile that may help promote enhanced adherence to oral antidiabetic therapy.

**Significant findings of the study:** In an Asian population, once-daily metformin extended-release (MXR) appears to have improved gastrointestinal tolerability, with fewer side-effects and discontinuations compared with prior oral antidiabetic (OAD) treatment. Treatment with MXR resulted in effective glycemic control in OAD-naïve patients and those switching from prior OAD therapy.

**What this study adds:** New short-term data on the efficacy, safety, and tolerability of MXR therapy in routine clinical practice in Asia, including data on the incidence of OAD-related side-effects and their impact on treatment discontinuation.

## Introduction

Implementing evidence-based measures to improve the prevention and management of type 2 diabetes mellitus (T2DM) is a paramount public health priority in Asia.^1^ A shift towards energy-rich diets and sedentary “obesogenic” modern lifestyles, brought about by increasing affluence and rapid urbanization, and population aging are contributing to the escalating prevalence of T2DM in the Asia–Pacific region.^1^–^4^ World Health Organization (WHO)^5^ figures project that the number of people with diabetes worldwide, 95% or more of whom have T2DM, will double by 2030 and that Asia–Pacific countries, which are home to more than half of the world’s population, will bear the greatest burden. Furthermore, because Asians are predisposed to developing T2DM at a younger age than non-Asians, they suffer from complications for longer and die earlier.^1^ Consequently, elevated rates of cardiovascular and cerebrovascular disease will incur substantial burdens of morbidity and mortality, healthcare costs, and lost income in the region.^1^,^2^,^5^,^6^ The WHO predicts that by 2015 deaths attributable to diabetes will increase by 39% and 51% in countries comprising the WHO South-East Asia^7^ and Western Pacific regions,^8^ respectively.

Conventional metformin therapy with immediate-release formulations has been a mainstay of T2DM therapy for more than 30 years.^9^,^10^ Immediate-release metformin (MIR) is most effective at an average dose of approximately 2000 mg/day,^11^ and has pharmacokinetic characteristics that typically require this to be divided between two or three smaller doses.^12^ Although this regimen is generally well tolerated, 20% or more of patients experience adverse gastrointestinal (GI) side-effects, most commonly diarrhea, nausea, and vomiting.^11^,^13^,^14^ Although these GI side-effects often diminish over time and can be minimized by careful dose adjustment and taking metformin at mealtimes,^15^,^16^ they may impair compliance and cause approximately 5% of patients to discontinue therapy.^9^,^17^ The need to take several tablets each day has also been shown to negatively impact compliance with oral antidiabetic (OAD) therapy.^17^–^21^ It is widely acknowledged that compliance to drug therapy is a crucial determinant of patient outcomes, and that drug regimens should be simplified as far as possible to support greater compliance.^9^

Absorption of MIR occurs predominantly in the upper GI tract,^22^ with peak serum concentrations achieved within 3 h. Although it remains unknown how metformin causes GI side-effects,^23^ extended-release metformin (MXR) formulations that delay time to peak metformin plasma concentrations and smooth plasma metformin peak and trough levels may lead to improved tolerability compared with MIR.^24^ An MXR formulation (Glucophage® XR; Merck Santé, Lyon, France) has been developed and studies have been performed to test the hypothesis that this may improve GI tolerability and enable once-daily dosing.^10^,^12^ A two-phase hydrophilic polymer matrix is used in the MXR, comprising an outer layer that hydrates to form a gel when exposed to fluid in the GI tract and a particulate inner phase from which metformin elutes gradually by diffusion over the dosing interval.^12^,^25^ In contrast with MIR, which releases 90% of the drug within 30 min, MXR has longer gastric residence and is absorbed more slowly from the upper GI tract, with 90% release over 10 h, which delays the time to peak concentrations by approximately 4–7 h.^12^,^13^,^25^ Taking MXR with the evening meal exploits naturally slower GI emptying post-prandially and at night to prolong absorption and permit once-daily dosing.^12^,^26^ Pharmacokinetic studies show that MXR once-daily has comparable overall bioavailability to an equivalent twice-daily dose of MIR, but that the steady-state 24-h plasma profile of the extended-release (XR) formulation has less pronounced peaks and troughs.^10^,^12^,^27^,^28^

Clinical studies in both predominantly Caucasian populations^25^ and in Chinese patients with T2DM^29^ have demonstrated that equivalent doses of MIR and MXR have similar antihyperglycemic efficacy. Further studies conducted in the US and UK have confirmed that among patients switched from MIR to comparable doses of MXR, the XR formulation has improved GI tolerability, with significantly fewer GI side-effects;^24^,^30^ glycemic efficacy in these studies was either equivalent or improved. By allowing once-daily administration, MXR can also simplify OAD treatment^9^ and has been demonstrated to significantly improve treatment adherence.^23^,^30^ However, this evidence and other data on MXR derive from predominantly Caucasian populations; given the magnitude of the T2DM epidemic in Asia, it is important that best clinical practices are based on robust data obtained from populations in which these practices will be applied.

There are few studies of MXR (which became available in the Asia–Pacific region in 2007) in Asian patients with T2DM, with the studies that have been performed generally small and providing sparse data on the incidence of GI side-effects.^4^ Prospective pan-Asian observational data may provide valuable information regarding the experience of using MXR in T2DM management among this patient population, as well as insights into the use of MXR in routine clinical practice in Asian countries. To this end, the present study was designed to test the hypothesis that, by enabling a simplified, once-daily dosing schedule and reducing the incidence of GI side-effects, MXR therapy may contribute to improved management of Asian patients with T2DM. The main objectives of the present study were to assess the tolerability of MXR in the routine clinical treatment of Asian patients with T2DM and its effectiveness in maintaining glycemic control.

## Methods

### Study design and patients

The present prospective observational study was conducted from January to December 2008 at hospitals and/or clinics in Hong Kong, Indonesia, Malaysia, the Philippines, Singapore, and South Korea (see Appendix I). Each study center enrolled consecutive patients who had a diagnosis of T2DM and who were prescribed MXR (Glucophage® XR; Merck Santé) therapy for T2DM. The study excluded patients who had been treated with MXR prior to the start date, those in whom MXR therapy was contraindicated by the local label, and those planning to continue therapy with another OAD during the study period. The study required no additional procedures, examinations, or other deviations from standard medical management, nor active involvement of the patients. The present study was approved by the institutional ethics committees of the respective country coordinating investigators.

### Data collection

#### Baseline visit

Baseline data collected anonymously when each patient was first prescribed MXR included gender, weight, height, age, duration of diabetes, history of OAD therapy, medical history (including underlying GI disease, other concurrent illnesses, and side-effects of previous MIR therapy), HbA1c level, and fasting glucose (when available). In cases in which the HbA1c test was not performed at the baseline visit, the most recent HbA1c reading before starting MXR therapy was recorded as the baseline value.

#### Post-treatment visit

Patients were followed-up for at least 12 weeks after commencing MXR therapy, or until treatment discontinuation if this occurred earlier than 12 weeks. In cases of premature discontinuation, the reason was recorded as inadequate glycemic control, patient request, GI side-effects, non-GI side-effects, or other.

Post-treatment data were collected at the clinic visit closest to the calculated Week 12 visit date or closest to when treatment was discontinued, if earlier, and included final MXR dosage, side-effects (if any) experienced during MXR therapy, HbA1c level, and fasting glucose (when available). In cases in which the HbA1c test was not performed at the post-treatment visit, the HbA1c reading taken closest to the calculated Week 12 visit date was recorded as the post-treatment value.

Patients who returned for the post-treatment visit were deemed to have completed the study; those who failed to return were recorded as “lost to follow-up”.

#### Safety reporting

Safety reporting was performed as required by MXR post-marketing surveillance.

### Study endpoints

The primary study endpoint was the proportion of patients discontinuing MXR treatment prematurely, defined as <12 weeks after starting, due to side-effects.

Secondary study endpoints were the proportion of patients remaining on MXR treatment for at least 12 weeks, the proportion of patients experiencing at least one GI side-effect, the incidence of side-effects, reasons for discontinuation, and changes in HbA1c levels from baseline after at least 12 weeks of MXR therapy.

### Data management and statistical analyses

Baseline and post-treatment data were entered into a case report form (CRF) at the visit hospital/clinic. Completed CRFs were collected from participating sites for centralized data validation and entry. Patients who did not complete the study were not replaced. Patients were excluded from an analysis if the relevant data were missing, but not from other analyses for which data were available.

The sample size was calculated based on the primary endpoint. From previous data,^9^ it was estimated that the proportion of patients who discontinue MXR treatment prematurely (i.e. <12 weeks) due to side-effects is 2.5%–5.0%. Based on a total of 2500 patients, the corresponding two-sided 95% confidence intervals are 1.9–3.1 for an expected rate of 2.5%, and 4.1–5.9 for an expected rate of 5%.

Separate subgroup analyses of the endpoints were performed according to patients’ study location and prior exposure to other OAD therapy.

The significance of differences between results was determined post hoc using appropriate standard statistical tests. Student’s *t*-test was used for continuous variables and the Chi-squared test was used for categorical variables. Unless indicated otherwise, data are given as the mean ± SD.

## Results

### Patient disposition and demographics

Participating hospitals/clinics enrolled 3592 patients, with most (87.7%) from South Korea. Of these, 3556 (safety population) received MXR treatment; 25 patients withdrew and 11 had no data for study completion. Post-baseline HbA1c assessments were available for 3471 patients treated with MXR (the intent-to-treat [ITT] population), of whom 23 withdrew and 22 had no data for study completion. A total of 3464 (97.4%) safety population patients completed 12 weeks of MXR treatment, 63 (1.8%) patients withdrew or dropped out during the study, and 29 had no data for study completion. Post-baseline HbA1c assessments were available for 3426 safety population patients who completed 12 weeks of treatment. There were no protocol violations.

The demographic characteristics of the ITT patient population are given in Table 1. The median age of patients in the ITT population was 57 years, ranging from 12 years (Indonesia) to 95 years (South Korea). The Singapore patient cohort (*n* = 19) had the youngest mean age of 48.8 ± 19.8 years, where the Hong Kong cohort (*n* = 15) had the oldest at 60.9 ± 14.5 years. The male:female ratio of the ITT population was approximately 1:1; however, this varied between countries. The mean weight and height of the patients were similar between countries, at approximately 67 kg and 163 cm, respectively.

**Table 1:** Patient demographics, by country and overall (intent-to-treat population)

	Hong Kong	Indonesia	Malaysia	Philippines	Singapore	South Korea	Total
No. patients	15	116	130	103	19	3088	3471
Age (years)							
*n* (missing)	15 (0)	116 (0)	129 (1)	102 (1)	19 (0)	3087 (1)	3468 (3)
Mean ± SD	60.9 ± 14.5	51.6 ± 11.4	50.3 ± 12.8	52.0 ± 12.3	48.8 ± 19.8	57.9 ± 11.1	57.2 ± 11.5
Median (range)	59 (39–84)	53 (12–79)	49 (18–85)	53 (18–80)	45 (14–89)	58 (22–95)	57 (12–95)
Sex							
No. men (%)	6 (40.00)	71 (61.21)	76 (58.46)	42 (40.78)	7 (36.84)	1525 (49.38)	1727 (49.76)
No. women (%)	9 (60.00)	45 (38.79)	54 (41.54)	60 (58.25)	12 (63.16)	1555 (50.36)	1735 (49.99)
No. missing (%)	0 (0.00)	0 (0.00)	0 (0.00)	1 (0.97)	0 (0.00)	8 (0.26)	9 (0.26)
Weight (kg)							
*n* (missing)	14 (1)	112 (4)	129 (1)	103 (0)	19 (0)	2939 (149)	3316 (155)
Mean ± SD	68.98 ± 15.53	68.44 ± 11.45	73.66 ± 17.79	68.75 ± 16.21	73.61 ± 16.44	66.69 ± 10.12	67.13 ± 10.96
Median (range)	66.5 (46.0–100.9)	68.0 (40.0–110.0)	72.0 (35.0–140.0)	69.4 (37.3–159.0)	70.1 (43.2–114.1)	67.0 (32.0–120.1)	67.0 (32.0–159.0)
Height (cm)							
*n* (missing)	14 (1)	112 (4)	126 (4)	102 (1)	17 (2)	2940 (148)	3311 (160)
Mean ± SD	160.1 ± 11.3	162.9 ± 7.7	161.7 ± 9.0	161.4 ± 8.2	162.4 ± 8.6	163.2 ± 8.5	163.0 ± 8.5
Median (range)	155 (146–180)	164 (145–182)	162 (142–186)	162 (145–179)	161 (151–181)	163 (138–187)	163 (138–187)

### History of diabetes and concurrent illness

The mean duration of T2DM in the ITT population at study entry was 2.8 ± 3.4 years (Table 2). The duration of T2DM differed between the six countries, with mean values lying between approximately 2 and 4 years in Indonesia, the Philippines, and South Korea, but at least two- to threefold longer, at approximately 7–8 years, in Hong Kong, Singapore, and Malaysia. Half (50.1%) of the ITT patient population had concurrent illnesses (Table 2), most commonly hypertension (39.5%), dyslipidemia (18.2%), obesity (7.1%), and coronary artery disease (4.3%), as defined according to criteria used by the enrolling physician. Patients in the Indonesian cohort had a lower prevalence of hypertension (12.1%) relative to the other countries; patients in Hong Kong had a comparatively higher prevalence of hypertension (93.3%), coronary artery disease (40.0%), and a high prevalence of dyslipidemia (53.3%). The highest prevalence of obesity was seen in patients from Malaysia (29.2%), Hong Kong (26.7%), and the Philippines (21.4%).

**Table 2:** History of diabetes, concurrent illness and oral antidiabetic therapy, by country and overall (intent-to-treat population)

	Hong Kong	Indonesia	Malaysia	Philippines	Singapore	South Korea	Total
No. patients	15	116	130	103	19	3088	3471
T2DM duration (years)							
*n* (missing)	7 (8)	56 (60)	90 (40)	64 (39)	9 (10)	2414 (674)	2640 (831)
Mean ± SD	8.44 ± 4.94	1.95 ± 2.83	7.99 ± 7.95	3.99 ± 4.22	6.85 ± 5.89	2.54 ± 2.81	2.78 ± 3.36
Median (range)	10.0 (0.1–16.0)	0.8 (0.1–15.0)	6.0 (0.2–50.0)	3.0 (0.0–21.0)	7.0 (0.1–19.0)	1.5 (0.0–30.4)	1.5 (0.0–50.0)
Concurrent illness*							
Yes	14 (93.33%)	48 (41.38%)	101 (77.69%)	72 (69.90%)	6 (31.58%)	1499 (48.54%)	1740 (50.13%)
Missing	1 (6.67%)	5 (4.31%)	15 (11.54%)	3 (2.91%)	4 (21.05%)	0 (0.00%)	28 (0.81%)
Hypertension	14 (93.33%)	14 (12.07%)	73 (56.15%)	49 (47.57%)	8 (42.11%)	1213 (39.28%)	1371 (39.50%)
Dyslipidemia	8 (53.33%)	41 (35.34%)	71 (54.62%)	41 (39.81%)	7 (36.84%)	462 (14.96%)	630 (18.15%)
CAD	6 (40.00%)	0 (0.00%)	13 (10.00%)	10 (9.71%)	1 (5.26%)	119 (3.85%)	149 (4.29%)
Obesity	4 (26.67%)	8 (6.90%)	38 (29.23%)	22 (21.36%)	0 (0.00%)	175 (5.67%)	247 (7.12%)
OAD therapy prior to MXR therapy							
Yes	11 (73.33%)	48 (41.38%)	94 (72.31%)	52 (50.49%)	13 (68.42%)	759 (24.58%)	977 (28.15%)
Missing	2 (13.33%)	10 (8.62%)	14 (10.77%)	5 (4.85%)	2 (10.53%)	12 (0.39%)	45 (1.30%)
MIR	9 (60.00%)	37 (31.90%)	99 (76.15%)	26 (25.24%)	14 (73.68%)	587 (19.01%)	772 (22.24%)
Others	6 (40.00%)	40 (34.48%)	59 (45.38%)	42 (40.78%)	9 (47.37%)	282 (9.13%)	438 (12.62%)
Prior OAD duration (years)							
*n* (missing)	7 (8)	37 (79)	97 (33)	26 (77)	14 (5)	574 (2514)	755 (2716)
Mean ± SD	2.83 ± 3.55	1.82 ± 1.46	4.60 ± 3.85	3.74 ± 3.43	3.10 ± 2.92	2.39 ± 2.48	2.71 ± 2.81
Median (range)	1.4 (0.1–10.0)	1.3 (0.3–5.4)	3.3 (0.1–15.0)	2.4 (0.3–15.0)	2.7 (0.1–10.0)	1.5 (0.0–20.1)	1.7 (0.0–20.1)
Final MIR dose before discontinuation (mg/day)							
*n* (missing)	10 (5)	41 (75)	100 (30)	29 (74)	15 (4)	586 (2502)	781 (2690)
Mean ± SD	1225.0 ± 767.8	902.4 ± 320.5	1600.0 ± 575.9	1158.6 ± 480.7	1123.3 ± 570.4	911.7 ± 344.9	1016.6 ± 460.0
Median (range)	1000 (500–3000)	1000 (500–1500)	1700 (500–3000)	1000 (500–2400)	1000 (250–2000)	1000 (2–2000)	1000 (2–3000)
Did patient experience side-effect/s during MIR therapy?							
Yes	0 (0.00%)	2 (5.41%)	3 (3.03%)	0 (0.00%)	0 (0.00%)	15 (2.56%)	20 (2.59%)
Missing	1 (11.11%)	0 (0.00%)	11 (11.11%)	3 (11.54%)	1 (7.14%)	2 (0.34%)	18 (2.33%)
GI side-effects	2 (22.22%)	14 (37.83%)	59 (59.59%)	4 (15.38%)	2 (14.29%)	245 (41.74%)	326 (42.23%)
Diarrhea	2 (22.22%)	0 (0.00%)	12 (12.12%)	1 (3.85%)	0 (0.00%)	156 (26.58%)	171 (22.15%)
Nausea	0 (0.00%)	2 (5.41%)	10 (10.10%)	0 (0.00%)	1 (7.14%)	79 (13.46%)	92 (11.92%)
Abdominal distention	1 (11.11%)	0 (0.00%)	36 (36.36%)	0 (0.00%)	0 (0.00%)	16 (2.73%)	53 (6.87%)
Dyspepsia	0 (0.00%)	12 (32.43%)	42 (42.42%)	1 (3.85%)	0 (0.00%)	17 (2.90%)	72 (9.33%)
Flatulence	0 (0.00%)	0 (0.00%)	11 (11.11%)	0 (0.00%)	1 (7.14%)	4 (0.68%)	16 (2.07%)
Constipation	0 (0.00%)	0 (0.00%)	2 (2.02%)	0 (0.00%)	0 (0.00%)	1 (0.17%)	3 (0.39%)
Abdominal pain	0 (0.00%)	0 (0.00%)	1 (1.01%)	2 (7.69%)	0 (0.00%)	2 (0.34%)	5 (0.65%)
Non-GI side-effects	0 (0.00%)	0 (0.00%)	2 (2.02%)	0 (0.00%)	0 (0.00%)	0 (0.00%)	2 (0.26%)
Was MIR discontinued due to side-effect/s?							
Yes	2 (22.22%)	1 (2.70%)	3 (3.03%)	2 (7.69%)	0 (0.00%)	196 (33.39%)	204 (26.42%)
Missing	1 (11.11%)	25 (67.57%)	43 (43.43%)	21 (80.77%)	12 (85.71%)	310 (52.81%)	412 (53.37%)

* Defined according to criteria of prevailing guidelines followed in each country.

Unless indicated otherwise, data show the number of patients in each group, with percentages given in parentheses.

CAD, coronary artery disease; GI, gastrointestinal; MIR, metformin immediate-release; MXR, metformin extended-release; T2DM, type 2 diabetes mellitus.

### Previous OAD therapy

Most ITT patients (70.6%) had not received OAD treatment previously (Table 2); however, this largely reflects the situation in South Korea (75.0% of patients had not had prior OAD treatment), which contributed the bulk of patients. In Indonesia and the Philippines, approximately half the patients had no prior OAD treatment, whereas in Hong Kong, Singapore, and Malaysia approximately 70% of patients had already been treated with OAD(s). Most patients who had received prior OAD therapy had been treated with MIR (772/977; 79.0%), for a mean treatment duration of 2.7 ± 2.8 years. Fewer than half of those with prior OAD therapy (438/977; 44.8%) had been treated with agents other than metformin.

### Treatment

The starting MXR dose in most safety population patients was either 250–500 mg/day (47.6% of patients) or 750–1000 mg/day (44.2% of patients); only 8.2% of patients (291/3556) received doses of 1250 mg/day or greater. Of the 3464 safety population patients who completed 12 weeks of MXR treatment, 368 (10.6%) had the dose changed or adjusted during the study. The dose was also changed or adjusted in 25.4% of patients (16/63) who either withdrew or dropped out before completing the 12-week treatment period. At study end, 41.6% of patients were on an MXR dose of 250–500 mg/day, 45.5% were on a dose of 750–1000 mg/day, and 12.9% were on a dose of 1250 mg/day or greater, indicating a slight shift towards higher doses during the 12-week study. Overall, mean dose exposure of the safety population was 840.4 ± 347.2 mg/day, for a mean duration of 98.3 ± 20.9 days, totaling a mean dose exposure of 82 429.4 ± 38 189.1 mg.

### Side-effects and discontinuations during MXR therapy

Few of the 3556 patients with T2DM treated with MXR or the 3464 (97.4%) patients who completed 12 weeks’ treatment experienced side-effects. Only 128 (3.6%) patients experienced side-effects during the study (Table 3), which predominantly affected GI function (118/128; 3.3%). The most common side-effects were diarrhea (1.0%), dyspepsia (0.7%), nausea (0.6%), and flatulence (0.5%). Side-effects were the cause of 24 (0.7%) premature treatment discontinuations (primary endpoint), 17 due to GI side-effects and seven due to non-GI side-effects (Table 3). Inadequate glycemic control caused discontinuation in only three (0.1%) instances.

**Table 3:** Side-effects and premature discontinuations during 12 weeks of metformin extended-release therapy, according to country and overall (safety population)

	Hong Kong	Indonesia	Malaysia	Philippines	Singapore	South Korea	Total
No. patients	26	124	161	108	19	3118	3556
Did patient experience side-effect/s during 12 weeks’ MXR therapy?							
≥1 GI side-effect/s	3 (11.54%)	4 (3.23%)	28 (17.39%)	14 (12.96%)	0 (0.00%)	69 (2.21%)	118 (3.32%)
Diarrhea	0 (0.00%)	0 (0.00%)	9 (5.59%)	5 (4.63%)	0 (0.00%)	23 (0.74%)	37 (1.04%)
Nausea	0 (0.00%)	0 (0.00%)	2 (1.24%)	5 (4.63%)	0 (0.00%)	15 (0.48%)	22 (0.62%)
Abdominal distention	0 (0.00%)	0 (0.00%)	5 (3.11%)	1 (0.93%)	0 (0.00%)	10 (0.32%)	16 (0.45%)
Dyspepsia	0 (0.00%)	3 (2.42%)	7 (4.35%)	1 (0.93%)	0 (0.00%)	14 (0.45%)	25 (0.70%)
Flatulence	0 (0.00%)	0 (0.00%)	3 (1.86%)	5 (4.63%)	0 (0.00%)	11 (0.35%)	19 (0.53%)
Constipation	2 (7.69%)	0 (0.00%)	0 (0.00%)	0 (0.00%)	0 (0.00%)	3 (0.10%)	5 (0.14%)
Abdominal pain	0 (0.00%)	0 (0.00%)	0 (0.00%)	0 (0.00%)	0 (0.00%)	3 (0.10%)	3 (0.08%)
Completed ≥12 weeks’ MXR therapy?*							
Yes	14 (53.85%)	112 (90.32%)	129 (80.12%)	100 (92.59%)	19 (100.00%)	3090 (99.10%)	3464 (97.41%)
Withdrew /dropped out	6 (23.08%)	5 (4.03%)	27 (16.77%)	4 (3.70%)	0 (0.00%)	21 (0.67%)	63 (1.77%)
Missing	6 (23.08%)	7 (5.65%)	5 (3.11%)	4 (3.70%)	0 (0.00%)	7 (0.22%)	29 (0.82%)
Reasons for discontinuation							
Side-effect/s	1 (3.85%)	0 (0.00%)	17 (10.56%)	1 (0.93%)	0 (0.00%)	5 (0.16%)	24 (0.67%)
GI side-effect/s	0 (0.00%)	0 (0.00%)	14 (8.70%)	1 (0.93%)	0 (0.00%)	2 (0.06%)	17 (0.48%)
Non-GI side-effect/s	1 (3.85%)	0 (0.00%)	3 (1.86%)	0 (0.00%)	0 (0.00%)	3 (0.10%)	7 (0.20%)
Patient request	0 (0.00%)	0 (0.00%)	2 (1.24%)	1 (0.93%)	0 (0.00%)	1 (0.03%)	4 (0.11%)
Inadequate glycemic control	1 (3.85%)	0 (0.00%)	0 (0.00%)	1 (0.93%)	0 (0.00%)	1 (0.03%)	3 (0.08%)
Lost to follow-up	2 (7.69%)	3 (2.42%)	4 (2.48%)	1 (0.93%)	0 (0.00%)	2 (0.06%)	12 (0.34%)
Others	1 (3.85%)	1 (0.81%)	2 (1.24%)	1 (0.93%)	0 (0.00%)	0 (0.00%)	5 (0.14%)
Missing	1 (3.85%)	1 (0.81%)	4 (2.48%)	1 (0.93%)	0 (0.00%)	12 (0.38%)	19 (0.53%)

* The denominator used to calculate percentages is the number of patients who received treatment.

Data show the number of patients in each group, with percentages given in parentheses.

GI, gastrointestinal; MXR, metformin extended-release.

Patients who had already been treated with OAD(s) before receiving MXR in the present study experienced significantly more GI side-effects (5.7% vs 2.3%; *P* < 0.0001) and non-GI side-effects (0.7% vs 0.1%; *P* = 0.0046) than those without prior exposure to OADs (Table 4). These GI and non-GI side-effects caused a significantly higher proportion of patients already exposed to OADs to withdraw or drop out prematurely compared with those who had no previous exposure (1.6% vs 0.2%; *P* < 0.0001).

**Table 4:** Side-effects and premature discontinuations during 12 weeks of metformin extended-release therapy, according to prior oral antidiabetic therapy (safety population)

	Prior exposure to OAD therapy* (*n *=* *1026)	No prior exposure to OAD therapy (*n *=* *2478)	Unknown exposure to OAD therapy (*n *=* *52)	Total (*n *=* *3556)
Did patient experience side-effect/s during 12 weeks’ MXR therapy?				
≥1 GI side-effect/s	58 (5.65)	56 (2.26)	4 (7.69)	118 (3.32)
Diarrhea	8 (0.78)	27 (1.09)	2 (3.85)	37 (1.04)
Nausea	7 (0.68)	15 (0.61)	0 (0.00)	22 (0.62)
Abdominal distention	10 (0.97)	6 (0.24)	0 (0.00)	16 (0.45)
Dyspepsia	18 (1.75)	5 (0.20)	2 (3.85)	25 (0.70)
Flatulence	10 (0.97)	9 (0.36)	0 (0.00)	19 (0.53)
Constipation	2 (0.19)	3 (0.12)	0 (0.00)	5 (0.14)
Abdominal pain	3 (0.29)	0 (0.00)	0 (0.00)	3 (0.08)
Others	9 (0.88)	2 (0.08)	1 (1.92)	12 (0.34)
Non-GI side-effects	7 (0.68)	3 (0.12)	0 (0.00)	10 (0.28)
Completed ≥12 weeks’ MXR therapy?†				
Yes	975 (95.03)	2449 (98.83)	40 (76.92)	3464 (97.41)
Withdrew/dropped out	34 (3.31)	23 (0.93)	6 (11.54)	63 (1.77)
Missing	17 (1.66)	6 (0.24)	6 (11.54)	29 (0.82)
Reasons for discontinuation				
Side-effect/s	16 (1.56)	6 (0.24)	2 (3.85)	24 (0.67)
GI side-effect/s	10 (0.97)	5 (0.20)	2 (3.85)	17 (0.48)
Non-GI side-effect/s	6 (0.58)	1 (0.04)	0 (0.00)	7 (0.20)
Patient request	1 (0.10)	2 (0.08)	1 (1.92)	4 (0.11)
Inadequate glycemic control	1 (0.10)	2 (0.08)	0 (0.00)	3 (0.08)
Lost to follow-up	3 (0.29)	6 (0.24)	3 (5.77)	12 (0.34)
Others	3 (0.29)	2 (0.08)	0 (0.00)	5 (0.14)
Missing	11 (1.07)	7 (0.28)	1 (1.92)	19 (0.53)

* Patients treated with oral antidiabetic (OAD) therapy before metformin extended-release (MXR) therapy.

† The denominator used to calculate percentages is the number of patients who received treatment.

Data show the number of patients in each group, with percentages given in parentheses.

GI, gastrointestinal.

### Changes from baseline in HbA1c and fasting glucose

Treatment with MXR improved measures of glycemic control in the present study. The mean change in HbA1c among patients treated with MXR for whom post-baseline HbA1c assessments were available (ITT population) was −0.8 ± 0.9% from a mean baseline value of 8.0 ± 1.4% (Table 5). The mean change in fasting glucose in the ITT population was −1.5 ± 2.0 mmol/L from a mean baseline value of 8.6 ± 2.2 mmol/L.

**Table 5:** Change from baseline to post-treatment visit in HbA1c and fasting glucose, according to location and prior exposure to oral anti-diabetic therapy (intent-to-treat population)

	HbA1c (%)				Fasting glucose (mmol/L)			
		
	Baseline		Change from baseline		Baseline		Change from baseline	
	*n* (missing)	Mean ± SD	*n* (missing)	Mean ± SD	*n* (missing)	Mean ± SD	*n* (missing)	Mean ± SD
Location								
Hong Kong	13 (2)	7.75 ± 2.02	13 (2)	−0.80 ± 1.91	13 (2)	7.98 ± 3.29	7 (8)	−1.51 ± 3.10
Indonesia	116 (0)	8.88 ± 2.33	116 (0)	−1.26 ± 2.17	82 (34)	9.02 ± 3.66	79 (37)	−1.25 ± 3.12
Malaysia	130 (0)	8.53 ± 1.99	130 (0)	−0.94 ± 1.60	119 (11)	8.72 ± 3.36	106 (24)	−1.69 ± 3.67
Philippines	100 (3)	8.43 ± 1.38	100 (3)	−1.51 ± 0.90	91 (12)	10.07 ± 4.05	84 (19)	−3.65 ± 3.67
Singapore	19 (0)	8.25 ± 3.45	19 (0)	−1.26 ± 3.01	11 (8)	8.25 ± 4.09	3 (16)	0.93 ± 1.15
South Korea	3085 (3)	7.97 ± 1.22	3085 (3)	−0.79 ± 0.76	1783 (1305)	8.48 ± 1.82	1621 (1467)	−1.40 ± 1.48
Prior exposure to OAD therapy								
Yes	971 (6)	7.69 ± 1.32	971 (6)	−0.60 ± 0.90	687 (290)	8.39 ± 2.67	599 (378)	−1.31 ± 2.39
No	2448 (1)	8.17 ± 1.33	2448 (1)	−0.93 ± 0.91	1379 (1070)	8.67 ± 1.90	1270 (1179)	−1.60 ± 1.68
Unknown	44 (1)	8.12 ± 1.78	44 (1)	−0.70 ± 1.60	33 (12)	8.57 ± 3.74	31 (14)	−1.20 ± 3.12
Total	3463 (8)	8.04 ± 1.35	3463 (8)	−0.84 ± 0.93	2099 (1372)	8.58 ± 2.22	1900 (1571)	−1.50 ± 1.97

OAD therapy, oral antidiabetic therapy.

Patients with no previous exposure to OAD(s) had higher baseline HbA1c (8.2 ± 1.3% vs 7.7 ± 1.3%) and fasting blood glucose (8.7 ± 1.9 vs 8.4 ± 2.7 mmol/L) than those with prior exposure to OADs (Table 5). The mean improvement in glycemic control following MXR treatment was significantly greater among patients without previous exposure to OAD therapy than in those with previous exposure: −0.9 ± 0.9% vs −0.6 ± 0.90%, respectively, for HbA1c (*P* < 0.0001); −1.6 ± 1.7 vs −1.3 ± 2.4 mmol/L, respectively, for fasting glucose (*P* <0.0020).

## Discussion

The present study provides valuable benchmark data on the incidence of OAD-related side-effects and their impact on treatment discontinuation in Asian populations.

The data from the present study demonstrate that therapy with once-daily MXR has a favorable safety profile in a population of Asian patients with T2DM, predominantly from South Korea. There appeared to be a reduced incidence of GI side-effects and a lower rate of side-effect-related discontinuations with MXR compared with prior OAD treatment, most commonly MIR, although it must be noted that the treatment periods were not comparable in terms of duration (12 weeks vs an average of 2.7 years). More than 97% of patients successfully maintained MXR therapy for at least 12 weeks, with fewer than 1% of patients discontinuing prematurely due to side-effects. Patients receiving MXR therapy also had improved measures of glycemic control at study end.

In considering these findings, we acknowledge certain limitations. First, interpretations of the present observational study are subject to the same constraints that apply to any other; in particular, MXR and MIR were not compared head-to-head in a randomized blinded design. It must also be acknowledged that the study duration of only 12 weeks limits the conclusions that can be drawn about tolerability. In addition, the sample size, although fairly large overall, had a high proportion of patients enrolled from South Korea, with much smaller cohorts from the other five countries. For this reason, caution should be exercised in extrapolating the findings Asia wide.

Nevertheless, it is generally acknowledged that observational studies can complement randomized controlled trials by providing a gauge of real-life effectiveness. Although these data may not fully reflect the situation in each country, they do represent a wide cross-section of Asian patients with T2DM receiving routine care in daily practice, and therefore provide valuable insights into the use of MXR for the management of T2DM in Asia. Despite considerable differences between the degree of economic development in the participating countries and the diversity of healthcare systems, consistent similarities in the responses to MXR therapy support the validity of the observations. However, variations between countries may highlight important issues relating to the practicalities of T2DM management.

The comparatively small numbers of patients with T2DM enrolled by sites other than South Korea make it impossible to determine the extent to which demographic differences (e.g. male:female ratios) between T2DM patients from the different countries have a genuine epidemiologic basis, or were simply due to chance. However, the longer duration of T2DM and higher proportion of patients already receiving OADs among study subjects in Singapore, Hong Kong, and Malaysia suggest that these sites treat a higher proportion of patients with well-established T2DM compared with Indonesia, the Philippines, and South Korea. This may also explain the relatively high incidence of concurrent illness seen in Malaysia and, in particular, the Hong Kong patient cohort. Importantly, the varying demographic characteristics of patients enrolled in the present study indicate that our overall findings on tolerability and efficacy of MXR therapy reflect a broad T2DM patient population typical of that seen in clinical practice.

Data on the incidence of GI side-effects of metformin in patients with T2DM from countries in the Asia–Pacific region are sparce.^1^ Previous studies have reported GI side-effect rates of 19% in Japanese patients taking 500–750 mg metformin/day and in 12% of Pakistani patients at a mean daily dose of 1333 mg metformin.^31^,^32^ Our study adds valuable new data on the incidence of GI side-effects with MIR and MXR therapy in six Asian populations, as well as on tolerability and treatment discontinuation of MXR in non-Caucasian patients with T2DM. As in Western populations, the side-effects of metformin were predominantly GI in nature, with diarrhea and nausea reported most commonly. Rates of GI side-effects during MIR therapy among patients enrolled in the present study ranged between 14.3% and 59.6% (median 30.0%), similar to, if not higher than, the range of approximately 20%–40% reported in studies of Western subjects.^11^,^17^,^30^ Rates of MIR discontinuation due to GI side-effects were lower overall (median 5.6%), again in line with published studies, but varied between countries. The high discontinuation rate in the South Korean cohort of 33.4%, compared with a figure of approximately 5% commonly cited for predominantly Caucasian populations, may reflect the fact that patients were targeted for recruitment to the present study of MXR because they had already shown susceptibility to MIR side-effects.

Similar to studies published previously,^10^,^23^–^25^,^29^,^30^ MXR appeared to be better tolerated than MIR and other OAD therapies by Asian patients with T2DM in the present study; in particular, a considerably higher proportion of patients experienced GI side-effects during MIR therapy (42.3%) than among the MXR safety population (3.3%), of whom only 24 of the 3556 patients (0.7%) discontinued for this reason. These figures compare favorably with reported incidences of GI side-effects and related discontinuations in other studies of MXR.^9^,^24^ The higher incidence in the present study of GI side-effects among patients treated previously with OADs likely reflects recruitment of patients who were switched to MXR because of GI sensitivity to MIR; yet even among this group, discontinuations were rare (∼1%). The overall completion rate of 97.4% indicates that, as in Western studies, improved GI tolerability was reflected in high rates of adherence to MXR therapy in Asian patients with T2DM.

By the end of the study, most patients in these Asian countries were receiving either 250–500 mg (45.5%) or 750–1000 mg (41.6%) of MXR once-daily, the shift towards higher doses reflecting dose titration during the 12-week study. With a total mean dose exposure of 82 429.4 mg, these safety and tolerability findings serve as a valuable addition to the body of data on the safety of MXR in Asian patients.

Numerous clinical trials conducted in Asian countries have confirmed that the antihyperglycemic efficacy and cardiovascular benefits of metformin firmly established in predominantly Caucasian populations are also observed in Asian patients with T2DM.^32^–^36^ Consistent with previous studies indicating that glycemic control with MXR is equivalent to MIR,^23^,^25^ improved GI tolerability of MXR in the present study was achieved without compromising glycemic efficacy. In fact, mean levels of both HbA1c and fasting blood glucose fell from baseline in all country cohorts (except fasting glucose in Singapore), irrespective of whether patients had received prior OAD therapy. The greater decline among patients who were OAD naïve from an initially higher baseline is not surprising because the greatest benefits of antidiabetic therapy are generally achieved during initial treatment immediately following diagnosis. Given the well-known importance of tight glycemic control in reducing long-term risk of diabetes complications,^37^ it is particularly encouraging that Asian patients already taking MIR and other OADs who were switched to MXR appeared to derive additional benefit.

### Conclusions

International guidelines for the pharmacotherapy of T2DM are based largely on evidence that derives from studies of predominantly Caucasian populations. Given the growing burden of T2DM in the Asia–Pacific region, it is important to verify that best clinical practice in the region is attuned to documented characteristics and responses of patient populations in these countries.

The present observational study of MXR therapy in Asian, predominantly South Korean, patients with T2DM provides important data and valuable insights into the efficacy, safety, and tolerability of MXR therapy in routine clinical practice. In particular, we confirm in a short-term study that in Asian, as well as Caucasian, populations, MXR taken once-daily has improved GI tolerability compared with MIR and other OADs taken over the long term, resulting in fewer GI side-effects and treatment discontinuations. Furthermore, MXR also affords effective glycemic control in both OAD-naïve patients with T2DM and in those switching from prior OAD therapy. Based on these results, it is hoped that MXR may help promote adherence to OAD therapy, resulting in improved clinical outcomes.

## Appendix I

Glucophage® XR (Merck Santé, Lyon, France) Asian observational study investigators.

**China:** King Loong Cheung, Private practice, Hong Kong; Pui Yin Lee, Private practice, Hong Kong; Bun Lap Wong, Private practice, Hong Kong; Kwok Wing Chan, Private practice, Hong Kong; Wai Suen Leung, Private practice, Hong Kong; Wai Kit Ching, Private practice, Hong Kong.

**Indonesia:** Askandar Tjokroprawiro, Dr. Soetomo General Hospital, Jawa Timur; John M.F. Adam, Dr. Wahidin General Hospital, Makasar; Slamet Suyono, Dr. Ciptomangunkusumo General Hospital, Jakarta; Ketut Suastika, Sanglah General Hospital, Denpasar, Bali; Hikamt Permana, Dr Hasan Sadikin General Hospital, Bandung; Laode Rote Tumada, Private practice, Tangerang; Budi Sugiarto, Private practice, Jakarta.

**Malaysia:** Radhakrishna Sothiratnam, Columbia Asia Hospital, Negeri Sembilan, Malaysia; Chen Sun Teh, Private practice, Johor; Foo Chun Yew, Private practice, Kuala Lumpur; Nor Azizah Aziz, Hospital Pulau Pinang, Penang; Siang Chai, Private practice, Perak; Chik Kiong Tee, Private practice, Johor Bahru; Zaitun Taib, Private practice, Kelantan.

**Philippines:** Ernesto Ang, Cardinal Santos Medical Center, San Juan, Manila; Mary Agnes Motril, Fort Bonifacio General Hospital, Makati City; Nessael Rozul, Ricardo Rodriguez District Hospital, San Fernando Pampanga; Mary Flor G. Ong, Health Link Multi Specialty Clinic, Iloilo City; Kevin Cimafrnca, SPC Medical Specialty Center, Cebu City; Jose Raymundo Carlos, Bulacan Medical Emergency Response Team, Poblacion Malolos City; Evelyn Gamallo, Visayas Community Hospital, Cebu City; Allisa Calderon, Manila Doctors Hospital, Manila; Jane Doctora, Riverside Medical Center, Bacolod City.

**Singapore:** Kevin Eng-Kiat Tan, Mount Elizabeth Medical Centre.

**South Korea:** Chul-Hee Kim, Soonchunhyang University Bucheon Hospital, Bucheon; Nae-Hee Lee, Soonchunhyang University Bucheon Hospital, Bucheon; Yoon-Hang Cho, University of Ulsan College of Medicine, Seoul; Hye-Sun Seo, Yonsei University College of Medicine, Seoul; Kyung-Ah Han, Eulji General Hospital, Seoul; Han-Jin Oh, Cheil General Hospital, Seoul.
